# Research Progress in Nanoparticle Inhibitors for Crude Oil Asphaltene Deposition

**DOI:** 10.3390/molecules29051135

**Published:** 2024-03-03

**Authors:** Shuangchun Yang, Chenhui Yan, Jiatie Cai, Yi Pan, Qiuju Han

**Affiliations:** 1 Department of Petroleum and Natural Gas Engineering College, Liaoning Petrochemical University, No.1, West Section of Dandong Road, Wanghua District, Fushun 113001, China; yangchun_bj@126.com (S.Y.); ych707591817@163.com (C.Y.); panyi_bj@126.com (Y.P.); 2 Department of Petrochemical Engineering College, Liaoning Petrochemical University, No.1, West Section of Dandong Road, Wanghua District, Fushun 113001, China; hanqj280@126.com

**Keywords:** nanoparticles, asphaltene, inhibitor, metal oxide nanoparticles, organic nanoparticles, inorganic nonmetal nanoparticles

## Abstract

Currently, the alteration of external factors during crude oil extraction easily disrupts the thermodynamic equilibrium of asphaltene, resulting in the continuous flocculation and deposition of asphaltene molecules in crude oil. This accumulation within the pores of reservoir rocks obstructs the pore throat, hindering the efficient extraction of oil and gas, and consequently, affecting the recovery of oil and gas resources. Therefore, it is crucial to investigate the principles of asphaltene deposition inhibition and the synthesis of asphaltene inhibitors. In recent years, the development of nanotechnology has garnered significant attention due to its unique surface and volume effects. Nanoparticles possess a large specific surface area, high adsorption capacity, and excellent suspension and catalytic abilities, exhibiting unparalleled advantages compared with traditional organic asphaltene inhibitors, such as sodium dodecyl benzene sulfonate and salicylic acid. At present, there are three primary types of nanoparticle inhibitors: metal oxide nanoparticles, organic nanoparticles, and inorganic nonmetal nanoparticles. This paper reviews the recent advancements and application challenges of nanoparticle asphaltene deposition inhibition technology based on the mechanism of asphaltene deposition and nano-inhibitors. The aim was to provide insights for ongoing research in this field and to identify potential future research directions.

## 1. Introduction

In the process of crude oil extraction, the thermodynamic equilibrium of asphaltene can be easily disrupted due to alterations in the external conditions, such as the temperature and pressure, or changes in the composition of the oil sample [1,2]. Asphaltene, which is the most structurally complex, dense, and polar component with high surface activity in crude oil, is prone to deposition during mining operations [3]. The deposition of asphaltenes in crude oil can negatively impact the recovery factor of crude oil extraction or production and may cause scaling and equipment blockage [4,5,6,7]. The precipitation of asphaltenes within the reservoir, for instance, alters the wettability of the rock. This process can also lead to formation damage and pore throat obstruction, thereby decreasing the oil content [8]. Simultaneously, during petroleum exploration and extraction, asphaltene continues to accumulate, resulting in the obstruction of well pipes, separators, and other peripheral equipment by asphaltene. This severely compromises the efficiency of oil production [9,10]. The deposition of asphaltenes in the ground oil pipeline limits the flow in the pipeline and even clogs the pipeline, which also reduces the efficiency of oil transportation [11,12]. Asphaltene is also adsorbed on the refining unit, which leads to coking, equipment corrosion, and catalyst pollution [13]. These examples show that asphaltene deposition in crude oil is a common phenomenon in the petroleum industry. For example, the United States, Canada, Norway, and other places have experienced severe asphaltene deposition [14,15]. So far, some research results were achieved in applying asphaltene inhibitors at home and abroad. However, due to the strong directivity of inhibitors, there are significant differences in the composition and properties of crude oil and asphaltene from different sources, and the applicable sedimentary inhibitors are also different.

In recent years, nanotechnology has been widely used in oil and gas exploration, reservoir characterization, drilling and completion, and enhanced oil recovery [16,17]. As the core of nanotechnology, nanomaterials refer to ultrafine materials with at least one dimension in the nanoscale range in three-dimensional space. Compared with conventional chemical agents, nanomaterials have significant performance differences in terms of the surface effect, rheology, mass transfer effect, transient phenomenon, and particle migration [18,19]. Due to the unique physical and chemical properties, such as the surface effect, volume effect, and quantum effect, nanomaterials have shown great potential in improving oil recovery. The primary mechanism of nanomaterials to improve oil recovery is the change in wettability, the decrease in oil–water interfacial tension, and the change in oil–water mobility [20,21]. Nanoparticles have good suspension and catalytic properties due to their large specific surface area and adsorption capacity, and have also been used by researchers to inhibit asphaltene deposition. Currently, asphaltene inhibitors can be categorized as follows: naturally occurring fatty acid organics, surface-active substances containing benzene rings, vegetable oil or petroleum-derived products, ionic liquids, and nanoparticle inhibitors [22]. Fatty acid organic inhibitors have a good inhibitory effect and are harmless to the environment, but the concentration is high [23]. Surface active agent inhibitors containing a benzene ring can alter interfacial properties and maintain asphaltene in a stable dispersed state, whereas certain nonionic surfactants are incapable of dissolving asphaltene [24].The vegetable oils and oil-processing products exhibit high thermal stability, low permeability, and inherent self-drying properties; however, their application scope remains limited, with scarce research studies conducted in this area [25]. The inhibitors based on ionic liquids exhibit remarkably low vapor pressure, a low melting point, excellent thermal stability, non-flammability, and favorable solubility in various solvents. However, the production scale and intricate synthesis mechanism of these ionic liquids render them more costly compared with commercially available asphaltene inhibitors [26,27,28]. Moreover, compared with traditional organic asphaltene inhibitors (such as sodium dodecyl benzene sulfonate and phenol), it has significant advantages. It is considered one of the most promising solutions for asphaltene deposition [29].

In recent years, the utilization of nanotechnology for mitigating asphaltene deposition has garnered attention from researchers. Guerrero-Martin et al. [30] comprehensively examined the utilization of nanotechnology in addressing asphaltene deposition, focusing on the sol–gel method, co-precipitation method, synthesis of functionalized nanoparticles, and interactions between asphaltene and nanoparticles. They emphasized that future research should prioritize intelligent and responsive nanoparticles, self-assembling nanoparticles, nanoparticles with self-healing capacity, and nanomaterials with detection and monitoring capabilities. Fuentes et al. [31] reviewed the conventional methods, such as acidizing and clay inhibitors used to slow down the migration of fine powder and the application of different nanoparticles in enhanced oil recovery, low-salinity water injection, and hydraulic fracturing. However, there is a scarcity of comprehensive descriptions from diverse perspectives on the efficacy of various nanoparticle-based asphaltene deposition inhibitors. In this paper, the mechanism of action of asphaltene nano-inhibitors is reviewed, and the latest research progress of different kinds of nanoparticles is introduced. First, the mechanism of asphaltene deposition in crude oil is introduced, and the influencing factors of crude oil stability are explored from the aspects of crude oil composition, pressure, and temperature. Second, the mechanism of inhibiting asphaltene deposition by nanomaterials is discussed for metal oxide nanoparticles, organic nanoparticles, and inorganic nonmetal nanoparticles. In addition, the interaction between nano-inhibitors and asphaltenes is described based on molecular simulation techniques. Finally, a comprehensive analysis is provided regarding the challenges and opportunities faced by nanomaterials in effectively mitigating asphaltene deposition.

## 2. Analysis of Nanoparticles Inhibiting Asphaltene Deposition

**Figure 1:**
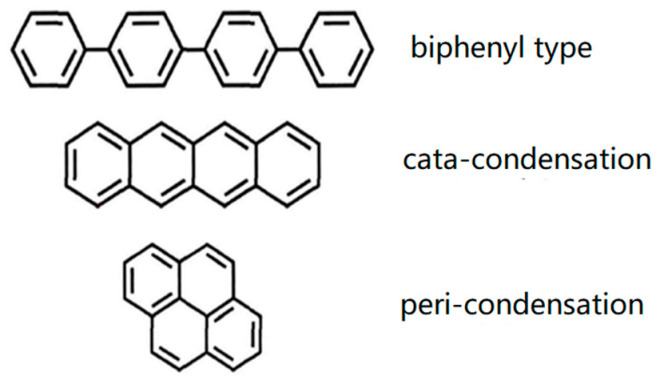
Condensation types of aromatic ring system.

Asphaltene is a black, glossy, and brittle solid with a molecular weight of 750~1200 Da. It comprises polycyclic aromatic hydrocarbon cores with fused rings and short alkyl chains. The common condensation methods of aromatic hydrocarbons are the biphenyl type, slim condensation, and forced condensation (as shown in Figure 1). The main components of asphaltenes are C and H. Most nonmetallic heteroatoms, such as N, O, and S, in crude oil and metal elements, such as Ni, V, Fe, and Ca, also exist in asphaltenes. The differences in molecular weight and structure of asphaltenes are primarily affected by the region in which they are located. Crude oil from different regions will lead to significant differences in the properties of asphaltenes. At the same time, the molecular weight of asphaltenes is also affected by extraction methods to a certain extent. For example, the average molecular weight of asphaltenes extracted with n-pentane is smaller than that of n-heptane.

**Figure 2:**
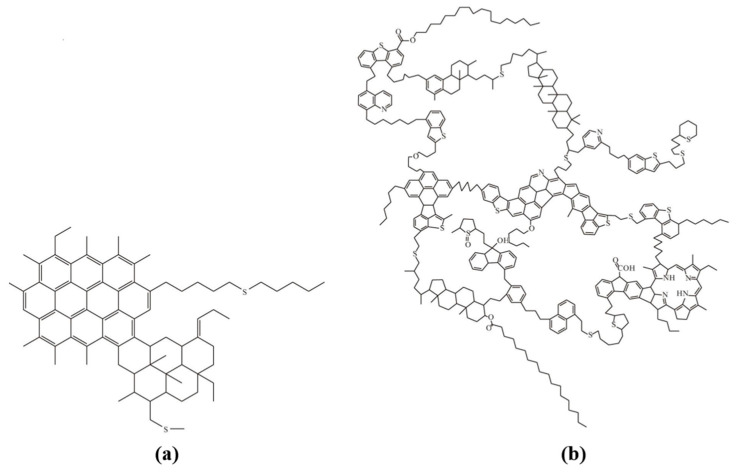
Isolated island model (**a**) and archipelago model (**b**) of asphaltene.

Although the molecular structure of asphaltene is exceedingly intricate and its precise characterization is challenging, numerous researchers conducted in-depth investigations on the analysis of asphaltene’s structure, resulting in the development of numerous distinct asphaltene structure models. Among them, the isolated island model and the archipelago model are the two most classic ones, as shown in Figure 2. The former was first proposed by Yen [32], and its structure contains polycyclic aromatic hydrocarbons, while the latter is composed of multiple polycyclic aromatic nuclei connected by alkyl bridges [33]. Subsequent researchers modified and supplemented their models based on these two models, and obtained several asphaltene structure models with different characteristics and properties, such as Mullins’ [34] Yen–Mullins model and Derek’s [35] improved Yen–Mullins model of benzodibenzothiophene based on the hydrocarbon asphaltene model.

### 2.1. Asphaltene Deposition Mechanism

In underground reservoirs, due to the particularity of external conditions and the presence of other light components and resins in crude oil, asphaltenes can be stably dispersed in crude oil. When crude oil is extracted from underground, due to the changes in temperature, pressure, and its components, the self-aggregation trend of asphaltenes will lead to the precipitation of asphaltenes from crude oil and the formation of deposits, which will lead to the blockage of pore throats in rock strata, the destruction of the pore structure, the reversal of reservoir wettability and a decrease in reservoir permeability, and changes in the physical and chemical properties of the crude oil. During the transportation process, asphaltene will cause a blockage of the pipeline, and the deposition of the downstream part will also cause a blockage of the pipeline. The acidic functional groups will also corrode the pipeline and production equipment, which is not conducive to actual production. Due to the frequent occurrence of asphaltene deposition in the production process of oil wells in a thriving area of Tahe Oilfield in China, the pipeline is blocked and punctured. The frequency of maintenance and cleaning is increased, which has a significant impact on the regular operation and productivity of oil wells, resulting in substantial economic losses [36].

Asphaltene deposition can be divided into three steps: instability, aggregation, and precipitation [37]. Asphaltene instability refers to the destruction of its equilibrium state to produce nanoscale asphaltenes; after that is the aggregation of asphaltenes, which means that the particles of asphaltenes can be observed at the microscale; the last stage is the precipitation process of the transition from stable nanoparticles to unstable micron particles.

#### 2.1.1. The Existing State and Influencing Factors of Asphaltenes

At present, the existing states of asphaltene in crude oil systems are mainly described by solution theory and colloid theory. Colloid theory is widely accepted.

Solution theory [38] refers to using asphaltene as a solute, a liquid crude oil group as a solvent, and a crude oil system as a homogeneous binary mixture. The solubility of asphaltene in crude oil is an essential factor that affects its dissolution. When asphaltene reaches saturation in crude oil, it will precipitate. The model based on this theory has a high accuracy in judging the precipitation point and precipitation amount of asphaltene. However, it ignores the complexity of a crude oil system and is not suitable for the prediction of a complex system.

Colloid theory [39] claims that crude oil is a colloidal system. The asphaltene in the crude oil colloidal system is used as a dispersed phase. The oil in the crude oil (saturates and aromatics) acts as a dispersed medium. The polarity and molecular weight of the crude oil are only lower than that of the asphaltene. The resin acts as a peptizer, and a relatively stable colloidal system is formed by the action of the resin and the dispersed medium. The crude oil colloid system is centered on asphaltene. The colloid molecules with larger molecular weight and aromaticity cover the surface of the asphaltene. The small molecules with relatively low aromaticity are attached around the colloid, and finally, transit to the intermicellar phase. However, there is no phase interface in the middle. Only when the characteristics and contents of each component of crude oil match each other can the asphaltene colloid system reach a stable state. In recent years, with the application of small-angle X-ray scattering (SAXS), small-angle neutron scattering (SANS), and ultra-small-angle X-ray scattering [40], it was confirmed that asphaltene exists in the form of micelles in crude oil.

**Figure 3:**
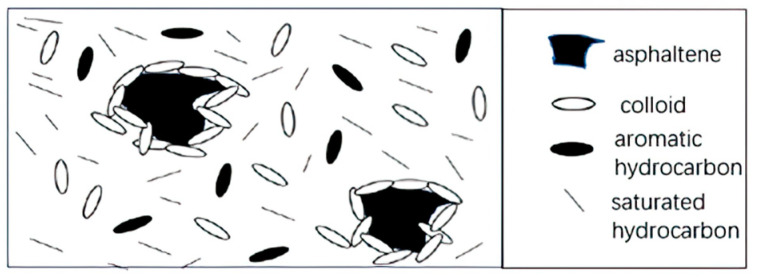
Asphaltene micelle solution.

For the colloid-based solution theory, scientists believe that asphaltenes dissolved in crude oil exist in a more stable dispersed colloid form, and crude oil is not a true solution. As shown in Figure 3, with asphaltene as the core, the resin is attached to its outside, which plays a good role in its stability. Some micelles composed of asphaltene and resin are only tens of nanometers, but some are relatively large and can even reach hundreds of nanometers.

Many studies showed that most crude oil exists in a relatively stable colloidal dispersion system under formation conditions, and asphaltenes are affected by many factors during deposition. Alian et al. [41] found that the stability of asphaltenes depends on the composition, pressure, and temperature of crude oil. Among them, the influence of constituent factors is greater.

**Figure 4:**
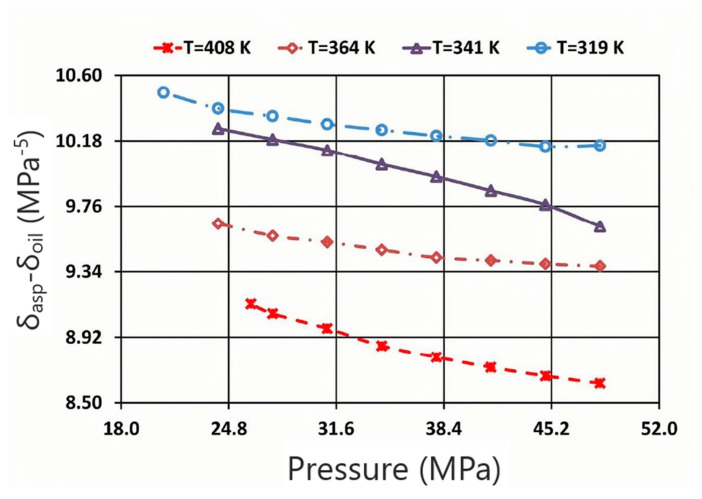
Diagram of oil–asphaltene solubility parameters of live oil samples with pressure and different fixed temperatures. Taken with permission from [42].

The effect of temperature on asphaltene deposition has different effects on different crude oils; therefore, it is necessary to judge according to the composition and properties of the crude oil system. Mohammadi et al. [42] designed a series of high-pressure–high-temperature decompression experiments to evaluate the effect of temperature on the precipitation and aggregation of asphaltenes in light oil, and they believed that the effect of temperature on the precipitation of asphaltenes was considerable. As the temperature of the decompression process increases, the amount of asphaltene precipitation decreases. As shown in Figure 4, the oil–asphaltene solubility parameter difference (δ_asp_-δ_oil_) decreases with increasing temperature (asphaltene solubility increases), resulting in a decrease in asphaltene precipitation. On the other hand, it is believed that the decrease in oil viscosity with temperature is the main driving force for the early precipitation of asphaltenes, rather than the dissolution of asphaltenes. In the light oil samples studied, the aggregation of asphaltenes at higher temperatures is controlled by the reaction-limited mechanism. The transition from reaction limited aggregation to diffusion limited aggregation in the mechanism of asphaltene aggregation occurred gradually as the temperature decreased.

Lu et al. [43] found that the effect of temperature on the stability of asphaltenes in the system is complex. When the temperature decreases, the change in cohesive energy density and evaporation internal energy increases the solubility of asphaltene in crude oil. However, as the temperature of crude oil increases, the density of crude oil becomes smaller, resulting in a weakening of the force between asphaltene and resin. Part of the resin desorbs from the surface of asphaltene, and asphaltene deposits due to Brownian motion aggregation and flocculation. The impact of temperature on asphaltene precipitation is multifaceted, involving the compositional and intrinsic properties of the crude oil. The effect of system pressure on asphaltene deposition in crude oil is also complicated. Some studies showed that the solubility of asphaltenes decreases with increasing pressure, and some studies found that the solubility of asphaltenes increases with increasing pressure [44]. Burke [45] found that asphaltene precipitation can only occur in a specific pressure range. If the pressure is lower or higher than this range, asphaltene precipitation will not occur. The bubble point pressure has a significant influence on asphaltene precipitation. Peyman et al. [46] designed a high-pressure unit and explored the effect of pressure on asphaltene deposition through experiments. It was found that when the pressure range was between 3 MPa and 14 MPa, the higher the pressure, the more asphaltene that was deposited.

Regarding component factors, Pu [47] found that when the content of the four components of resin, asphaltene, alkane, and aromatic hydrocarbon in the crude oil system was in a specific proportion range, the crude oil system was in a stable state. Li [38] also mentioned that asphaltenes and alkanes are not conducive to the stability of crude oil systems. At the same time, resins and aromatic hydrocarbons are conducive to the stability of crude oil systems.

In order to study the stability of asphaltene deposition more accurately, Cruz et al. [48] used a changing chamber equipped with a near-infrared probe to study the process of asphaltene deposition caused by carbon dioxide and evaluated the effects of pressure, temperature, asphaltene concentration, and system composition on asphaltene deposition. The asphaltene deposition process was simulated using the cubic positive correlation method. The results show that the temperature and oil model system composition are the main parameters that affect the stability of asphaltene. However, some people think that the resin also affects the stability of asphaltene. When the resin content is high, the asphaltene is not easy to precipitate, and when the resin content is low, the asphaltene becomes unstable and easy to precipitate [49]. Yena et al. [50] proposed to use the colloid instability index (CII) (the ratio of asphaltenes and saturated hydrocarbons to the content of aromatic hydrocarbons and resins) to measure the instability of asphaltene molecules. Although the CII is an empirical formula based on oilfield data, it can be used as a preliminary screening tool to identify possible precipitation problems in a crude oil system. When the CII is less than 0.7, asphaltene is considered stable, and when the CII is greater than 0.9, asphaltene is considered unstable. Zou [51] found that with the increase in system pressure, concentration difference, and molecular diffusion, the resin around the asphaltene become dispersed, which reduces the repulsive force between asphaltene micelles and the ability of the oil phase to carry asphaltene. Zhou [52] found that polar solid asphaltene micelles attract each other. When the gravitational force is greater than the buoyancy force, sedimentation occurs. When the pressure is greater than the minimum miscibility pressure, a single-phase state is formed. The increase in pressure and density makes the system more stable, and the precipitation of asphaltene decreases.

#### 2.1.2. Accumulation and Deposition of Asphaltenes

Researchers studied the aggregation and precipitation behavior of asphaltenes by various experimental methods, such as X-ray scattering and small-angle neutron scattering. Studies confirmed that asphaltenes are dispersed in crude oil as nanoparticles in the natural state [53].

When dissolved in organic solvents, asphaltene molecules can complex with each other to form different layers of polymers. The critical nano-aggregate concentration (CNAC) of asphaltene in toluene solution was measured by high-quality ultrasound. The experimental results show that when the asphaltene concentration is lower than the CNAC, the asphaltene molecules in toluene are dispersed into the real solution. When the asphaltene concentration is higher than the CNAC, the molecules stick together to form nano-aggregates or nano-molecules. The concentration of asphaltenes from real molecular solutions to nano-aggregates is deficient (mass fraction is about 10^−4^), and the number of asphaltene monomers in aggregates is very minimal [54]. DC conductivity measurements also confirmed the results of high-quality ultrasound studies. By comparing the changes in conductivity when the concentration was lower or higher than the CNAC, it was shown that the aggregation number of asphaltenes may be very small (<10) [55]. In order to confirm this result, NMR measurements observed changes in rotational relaxation at the CNAC [56]. Centrifugal tests also showed that asphaltenes precipitated above the CNAC increased [57,58].

Other analyses were undertaken, such as small-angle neutron scattering, Langmuir–Blodgett film studies, atomic force microscopy measurements, and surface compression analysis of asphaltenes in toluene. It was also confirmed that there are about ten asphaltene monomers in nanoscale asphaltene aggregates in toluene solution [59,60]. Once nano-aggregates are formed between asphaltenes, and the concentration of asphaltenes in the solution is much larger than the critical nano-aggregate concentration (CNAC), a secondary aggregation process will occur between asphaltenes.

This stage is usually called the flocculation of nano-aggregates to form asphaltene nanoclusters. Photon correlation spectroscopy shows a critical cluster concentration in the secondary aggregation process. At this critical cluster concentration, the flocculation kinetics change significantly [61]. The DC conductivity experiment of asphaltene solution confirmed that the fracture of the conductivity curve can be observed at the critical cluster concentration of asphaltene [55]. This is also consistent with the flocculation determination of asphaltene by near-infrared spectroscopy [62].

**Figure 5:**
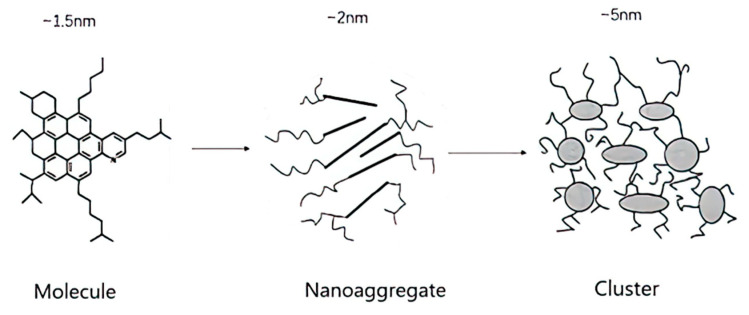
Yen–Mullin model of asphaltene coalescence flocculation.

American scientist Mullins has done much research on the structure of asphaltene [34,63,64]. Based on the research progress in the molecular weight, molecular structure, and aggregation characteristics of asphaltene, the asphaltene is described more accurately, and the model proposed by Yen was improved to provide a more accurate asphaltene structure, namely, the Yen–Mullin model, as shown in Figure 5. The graded asphaltene aggregation model in the improved Yen–Mullin model is a typical theoretical model of the asphaltene aggregation–flocculation process, which is generally accepted at present. In this model, six asphaltene molecules coalesce to form asphaltene nano-aggregates, and asphaltene nano-aggregates further flocculate to form asphaltene clusters. The asphaltene cluster contains eight asphaltene nanoclusters. According to the aggregation flocculation theory of asphaltenes, asphaltenes in the solution first forms polymers through intermolecular interactions. When the size and quantity of the polymer in the system reach the critical condition, the polymer further combines to form flocs.

Hu [65] employed molecular dynamics simulations to investigate the interaction between asphaltene aggregates and various solvents under constant temperature and pressure, aiming to simulate the carbon dioxide injection process. The results revealed that prior to CO_2_ injection, asphaltenes were uniformly dispersed within light components, such as methane, ethane, and octane; however, following the CO_2_ injection, mutual solubility was observed between the CO_2_ and light components, excluding asphaltenes, leading to asphaltene precipitation. This was attributed to the non-polar nature of CO_2_, which enhances both the hydrogen bonding and dipole interactions within asphaltene aggregates, while also significantly amplifying π-π charge transfer interactions. Consequently, an increased presence of aromatic clusters and heteroatoms in asphaltenes leads to a more pronounced deposition phenomenon upon carbon dioxide injection. Fang et al. [66] arrived at a similar conclusion using the same methodology, demonstrating that carbon dioxide gradually dissolves saturated hydrocarbons and resins, leading to the formation of asphaltene deposition.

Salehzadeh et al. [67] investigated the aggregation and adsorption/deposition behavior of asphaltene samples extracted from light, medium, and heavy oils. The findings revealed that asphaltenes and subcomponents with higher aromaticity exhibited enhanced tendencies for aggregation and adsorption/deposition, whereas lower aromaticity and longer side chains contributed to improved steric hindrance. The investigation of three distinct sub-components of medium-oil asphaltenes revealed that the stabilization of asphaltene sub-components with higher solubility resulted in a reduction in both aggregation and adsorption rates of asphaltenes with lower solubility.

### 2.2. Mechanism of Nano-Asphalt Inhibitor

**Figure 6:**
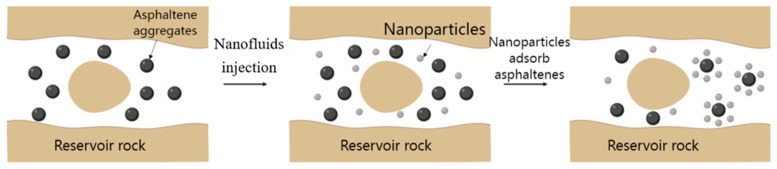
Schematic diagram of inhibiting asphaltene deposition by nanoparticle adsorption. Taken with permission from [70].

The strong polarity of the asphaltene surface is the fundamental reason for the self-association and even deposition of asphaltene molecules. Under the action of nanoparticles, asphaltene can use its strong polarity to inhibit asphaltene deposition and stably disperse in the crude oil system. On the one hand, by modifying the nanoparticles, the charge distribution on the surface of the particles can be changed, and the particles can adsorb asphaltenes after entering the reservoir, avoiding the aggregation and deposition of asphaltenes; on the other hand, nanoparticles can be grafted onto the surface so that the surface of the particles has a corresponding polar group. Therefore, after entering the reservoir, it will interact with asphaltene molecules to promote the stable dispersion of asphaltene. The adsorption of nanoparticles will inhibit the deposition process of asphaltene (Figure 6). After the injection of nanofluids, asphaltenes will be adsorbed by nanoparticles and stably dispersed in the crude oil system [68,69].

Nanoparticles can adsorb asphaltenes in crude oil and then be adsorbed on the surface of porous media, thereby delaying the flocculation behavior of asphaltenes with pressure, temperature, and composition changes, and changing their surface wettability according to the wetting preference of nanoparticles [70,71,72]. Mohammadi [73] found that 80% TiO_2_ nanocomposites had a higher stability and surface area than TiO_2_ nanoparticles. Hence, the adsorption of asphaltene on the surface of the particles increased, which increased the occurrence of asphaltene flocculation and was difficult to deposit. The adsorption process of nanoparticles on asphaltene is affected by many factors, such as the contact time, water content, temperature, and other molecules. Nassar et al. [74] found that the adsorption rate, affinity, and adsorption capacity of nanoparticles to asphaltenes depend on the relative molecular mass of asphaltenes. The smaller the relative molecular mass of asphaltenes, the greater the adsorption rate and the greater the adsorption capacity. The adsorption process of asphaltene on nanoparticles occurs in a relatively short time, and the magnetism of metal nanoparticles plays a vital role in the adsorption of asphaltene. In addition, pure asphaltene has a more robust adsorption capacity than asphalt or soft asphalt, which indicates that the degree of adsorption is affected by the chemical properties, molecular size, and structure of the crude oil components.

The adsorption model of nanoparticles on asphaltenes is still controversial. At present, the most widely accepted are the Langmuir adsorption model and the Freundlich adsorption model [75]. The Langmuir adsorption model represents a uniform monolayer adsorption. Although the asphaltene aggregates are heterogeneous, it is a reasonable and practical model for the dense layer of asphaltene aggregates with low concentration adsorption. Studies showed that the equilibrium adsorption data are in good agreement with the Langmuir model, confirming the rationality of the model [74,76]. The Freundlich adsorption model simulated the multi-layer adsorption structure of the heterogeneous surface. Nassar et al. found that the experimental data were significantly different from the calculated values of the model when using the Langmuir adsorption model for data fitting. Then, the Freundlich model was used to adjust the experimental data of the asphaltene adsorption isotherm. It was found that the data were in good agreement, indicating that the asphaltene was adsorbed to the heterogeneous surface through multi-layer adsorption [71,77].

The self-association between asphaltene molecules will accelerate the flocculation and deposition of asphaltene. According to the colloid theory, the dispersion effect of nanoparticles on asphaltenes is mainly due to the formation of a more stable interaction force between nanoparticles and asphaltene molecules or the formation of steric hindrance, thereby destroying the self-association between asphaltene molecules, thereby achieving the effect of inhibiting asphaltene deposition and removing asphaltene aggregates. Setoodeh et al. [78] synthesized Fe_3_O_4_ magnetic nanoparticles by the co-precipitation method. PT, MOF, GO, SiO_2_, and chitosan coatings were modifiers to enhance their adsorption properties for asphaltenes. It was found that the magnetic nanoparticles coated with polythiophene (PT) had better inhibition performance for asphaltene deposition in crude oil. This is because there are multiple sulfur heterocycles in the PT structure, which makes PT have higher polarity and can interact with N, S, and O heteroatoms in asphaltene molecules. Compared with other coated and uncoated nanoparticles, polarity, electrostatic, or van der Waals interactions lead to the enhanced adsorption of asphaltenes.

## 3. Asphaltene Nano-Inhibitors

Asphaltene deposition will inevitably occur in wellbores, production facilities, and transportation facilities, and corresponding measures must be taken to remove the deposited asphaltene. Before deposition, adequate measures need to be taken to avoid the precipitation of asphaltenes as much as possible. The methods of cleaning asphaltene deposition include physical removal, heat treatment, the chemical solvent method, the microbial method, and external force. The methods to prevent asphaltene precipitation are mainly various asphaltene dispersants/precipitation inhibitors.

Nanomaterials refer to solid materials with at least one dimension in the range of 0.1~100 nm in three-dimensional space, and are known as new materials across the century [79]. As early as 1959, American physicist Richard Feynman proposed that if the arrangement of materials on a small scale could be controlled, many substances with unique physical and chemical properties could appear. The natural synthesis of nanomaterials occurred in the 1980s. Professor H. Gleiter from Saarland University in Germany and Dr. Siegl from Argonne Laboratory in the United States successfully synthesized nanomaterials, which attracted wide attention. At the nanometer scale, the performance of particles is entirely different from that of large-scale particles [80]. The classical theory is no longer practical, and various special properties are possible.

In recent years, researchers around the world have carried out research on the use of a variety of nanomaterials to explore the inhibitory effect of nanoparticles on asphaltene deposition in reservoirs under different conditions and the influence of nanoparticle types on the inhibitory effect. At present, the nanoparticles used in the direction of enhanced oil recovery are mainly divided into three categories: metal oxide nanoparticles, organic nanoparticles, and inorganic nonmetal nanoparticles [81].

### 3.1. Metal Oxide Nanoparticles

Metal oxide nanoparticles, which are typically composed of one or more metal ions and oxygen ions, possess unique properties, such as high crystal structure, surface energy, and activity. Due to the low ionization potential and electronegativity of metal elements, the surface of metal oxides exhibits strong polarity. With the advancement of nanotechnology in recent years, metal oxide nanoparticles have been widely applied in various fields. The researchers found that metal oxides, such as Co_3_O_4_, NiO, and Fe_3_O_4_, have good catalytic ability, which can catalyze the decomposition of asphaltene [82,83]. Substances such as asphalt are adsorbed on the nanoparticles, disintegrate the viscoelastic network, and finally catalyze the formation of H_2_S, CH_4_, CO_2_, H_2_, and other substances, reducing the content of asphaltene in heavy oil. Iron oxide, Al_2_O_3_, NiO, ZnO, and other nanoparticles have also been used in the field of inhibiting asphaltene deposition, and some research results were achieved. It was found that metal oxide nanoparticles inhibited asphaltene deposition, mainly through adsorption [84].

Mercado et al. [85] investigated the catalytic and adsorption properties of five transition metal oxide nanoparticles, namely, MnO_2_, MoO_3_, NiO, Fe_2_O_3_, and V_2_O_5_, in the low-temperature oxidation (LTO) of asphaltenes. The results showed that the five nanoparticles all showed a certain catalytic line, which promoted low-temperature oxidation, among which Fe_2_O_3_, MnO_2_, and MoO_3_ performed better. The author posited that the redox properties were substantiated as correlated parameters for promoting the oxygen addition reaction in low-temperature oxidation processes. Simultaneously, these five nanoparticles also demonstrate exceptional asphaltene adsorption characteristics. Furthermore, it is considered that the oxidation activation energy of heavy oil in the LTO region exhibits an inverse relationship with the Langmuir affinity constant; specifically, a higher asphaltene/nanocatalyst affinity corresponds to a lower activation energy.

**Figure 7:**
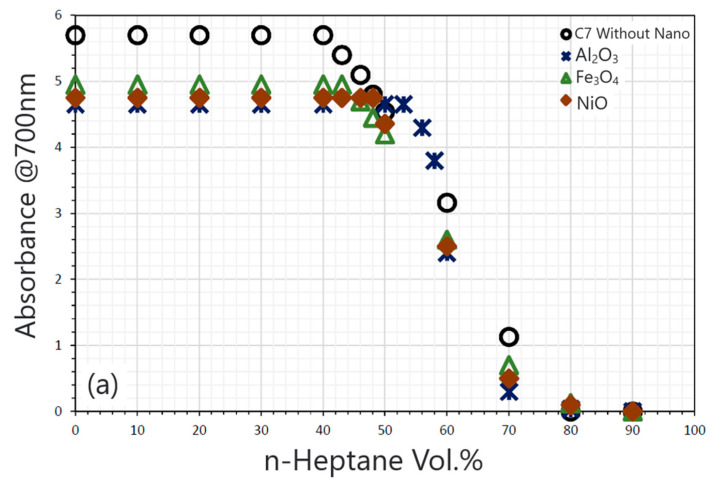

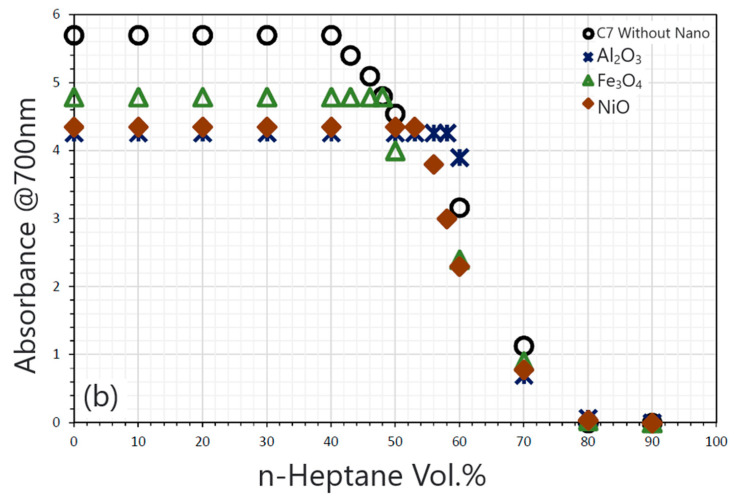
(**a**) The 0.01 wt% and (**b**) 0.1 wt% metal oxide nanoparticles were used to determine the starting point of synthetic oil containing 0.5 wt% asphaltene. Taken with permission from [88].

Mohammadi et al. [86] found that rutile nanoparticles (TiO_2_) can effectively improve the stability of asphaltenes in acidic media (pH < 4). However, in an alkaline environment, these nanoparticles cannot prevent the deposition of asphaltenes. Lu et al. [87] found that the injection of Al_2_O_3_ nanofluids can inhibit the deposition of asphaltenes on the surface of sand bodies in porous media, thereby reducing the permeability of crude oil. A 0.5 wt% nanoparticle concentration and 0.1 nanofluid/CO_2_ slug volume ratio are considered the optimum for inhibiting asphaltenes damage during CO_2_ flooding. Shojaati et al. [88] showed that the acidic nanoparticles γ-Al_2_O_3_ and NiO have better performance inhibiting asphaltene deposition than amphoteric nanoparticles Fe_3_O_4_, which can be seen in Table 1 and Figure 7. This is because acidic metal nanoparticles can polarize with asphaltene molecules, and the polar interaction between acidic nanoparticles and asphaltene is stronger than that of amphoteric nanoparticles. At the same time, compared with NiO nanoparticles, γ-Al_2_O_3_ has better performance. The author believed that acidic nanoparticles with Bronsted acid sites have more vital polarity and a stronger ability to form bonds with asphaltene molecules, and thus, the effect of inhibiting asphaltene deposition is better.

Iron oxide nanoparticles have attracted extensive attention due to their superparamagnetism, easy stability, and easy surface functionalization [89]. These kinds of known magnetic molecules mainly include “Fe_3_O_4_ magnetite, Fe^II^Fe^III^_2_O_4_, ferromagnetic, superparamagnetic (Size < 15 nm), α-Fe_2_O_3_ (hematite, weakly ferromagnetic or antiferromagnetic), γ-Fe_2_O_3_ (maghemite, ferrimagnetic), FeO (wüstite, antiferromagnetic), ε-Fe_2_O_3_ and β-Fe_2_O_3_”. Strong paramagnetic compounds have more prominent adsorption, which is due to the strong electron pairing tendency caused by the spin of unpaired electrons.

Kazemzadeh et al. [90] studied the effect of Fe_3_O_4_ nanoparticles on asphaltene precipitation by an IFT experiment. From Table 2, it can be seen that in the solution containing B-type asphaltene, the inhibitory effect of nanoparticles on asphaltene precipitation is more evident than that in the solution containing N-type asphaltene, indicating that the performance of nanoparticles varies significantly with the type of asphaltene in the solution. The author believed that this is because the nitrogen content of B-type asphaltene is greater than that of N-type asphaltene, and the H/C ratio is smaller than that of N-type asphaltene. That is, a low H/C ratio and high nitrogen content are beneficial to the adsorption of asphaltene on the surface of nanoparticles; the higher the mass fraction of Fe_3_O_4_ nanoparticles, the lower the strength of asphaltene deposition. However, Nassar et al. [91] found that metal oxide nanoparticles show strong affinity and catalytic activity for the adsorption and oxidation of asphaltenes. The interaction between the adsorbate and the adsorbent was used as the adsorption constant, and the order was NiO > Co_3_O_4_ > Fe_3_O_4_. The affinity constant of NiO was the highest, that is, it produced a stronger interaction, and thus, NiO exhibited the highest catalytic rate. The adsorption of Fe_3_O_4_ was the lowest, showing the lowest catalytic activity.

Although most of the recent research focused on a single nanoparticle, polymer-functionalized metal oxide nanoparticles have attracted more and more attention in recent years. Researchers synthesized NiO/SAPO-5 [92], NiO/ZSM-5 [93], Fe_3_O_4_-polythiophene [94], carboxylate-aluminoxane [95], and other composite nanoparticles, and found that they exhibit more excellent performance in inhibiting asphaltene.

**Figure 8:**
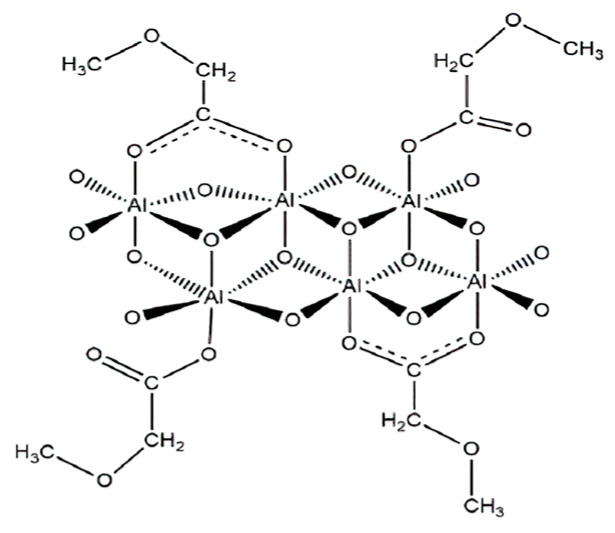
Molecular structure of carboxylate-alumoxane nanoparticles.

Bagherpour et al. [95] prepared two kinds of carboxylate–alumoxane nanoparticles using boehmite and pseudo-boehmite as raw materials. The structure is shown in Figure 8. The results demonstrate that with the increase of nanoparticle concentration, the adsorption efficiency of asphaltene also increases due to the existence of more adsorption sites, but the increase of nanoparticle number will reduce the adsorption capacity of a single nanoparticle. Nanoparticles with a large specific surface area and pore volume and small size have better adsorption capacity, higher adsorption efficiency, and better inhibition performance. Through the Freundlich isothermal curve, it was found that the carboxylate–alumoxane nanoparticles delayed the starting point of asphaltene precipitation, which helps to reduce the amount of asphaltene precipitation. At the same time, the author studied the mechanism of the adsorption phenomenon, which was attributed to molecular interactions, including hydrogen bonding, Lewis acid–base interaction, π-π interaction, and hydrophobic interaction.

Setoodeh et al. [94] compared the adsorption of asphaltene on Fe_3_O_4_ nanoparticles and Fe_3_O_4_ polythiophene nanocomposites and found that the adsorption capacity of the magnetite surface can be improved by coating polythiophene on the surface of nanoparticles. The adsorption rates of asphaltene on nanoparticles and nanocomposites were 65.94% and 78.98%, respectively. Tazikeh [96] discussed the inhibitory effect of polythiophene-coated Fe_3_O_4_ nanoparticles on the asphaltene precipitation and surface morphology. Atomic force microscopy imaging technology was used to obtain surface morphology information, and the effect of nanoparticles on the surface morphology was evaluated by double fractal and double Gaussian theory. This study found that the morphology of the asphaltene surface changed after the addition of nanoparticles. In the case of heavy synthetic oil, more asphaltenes were adsorbed on the nanoparticles. The author believed that in the case of heavy synthetic oil, multi-layer asphaltenes (chemisorption or adsorption type III) were formed, and more asphaltenes were adsorbed on the nanoparticles. Rezvani et al. [97] also tried to compound chitosan, TiO_2_, SiO_2_, and Fe_3_O_4_ nanoparticles to prepare Fe_3_O_4_-based nanocomposites and carried out asphalt adsorption tests. The results were Fe_3_O_4_/chitosan > Fe_3_O_4_/TiO_2_ > Fe_3_O_4_/SiO_2_ > Fe_3_O_4_. The composite material had a significant effect on inhibiting asphaltene deposition.

Scientists found that Co_3_O_4_ nanoparticles, which are also superparamagnetic, exhibit better asphaltene adsorption than Fe_3_O_4_ nanoparticles, which can significantly reduce interfacial tension and minimum miscibility pressure [98]. Meanwhile, in their investigation of Co_3_O_4_’s inhibition of asphaltene deposition, Wang et al. [99] synthesized Co_3_O_4_-SiO_2_ nanoparticles coated with a SiO_2_ film using the sol–gel method. It was observed that the presence of pores larger than 10 nm on the surface of Co_3_O_4_-SiO_2_ nanoparticles decreased significantly when compared with pure Co_3_O_4_ nanoparticles. Conversely, there was a notable increase in narrow mesopores (<10 nm). Additionally, the specific surface area increased from 20.87 m^2^/g to 29.25 m^2^/g, while the saturated adsorption capacity rose from 1.24 mg/m^2^ to 1.53 mg/m^2^.

### 3.2. Organic Nanoparticles

In this article, “organic” refers to the traditional definition of a compound containing carbon in all structures. Carbon is the sixth element in the periodic table of elements and one of the most abundant elements in the universe. This black powder comprises spherical nanoparticles with unique properties, which are usually synthesized by hydrothermal processes. These particles can be surface modified as expected, combining organic molecules or polymers onto the surface of the particles.

**Figure 9:**
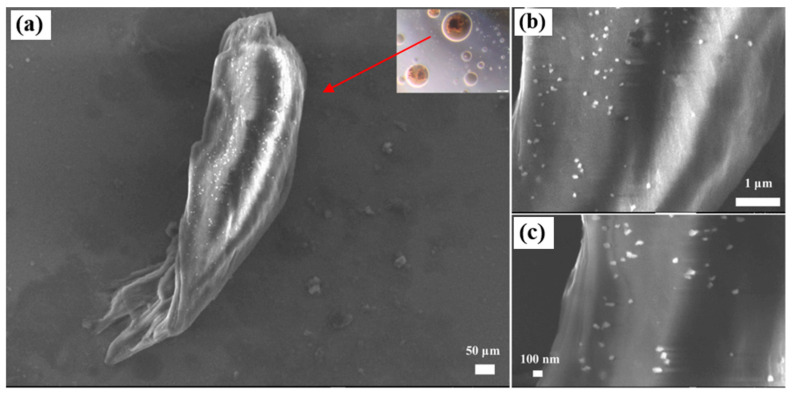
(**a**) SEM image of an agglomerated structure of the precipitate. Asphaltene nano-aggregates are seen to be adsorbed on the surface of GO. (**b**,**c**) Zoomed in images of the agglomerate. Taken with permission from [101].

Gandomar et al. [100] experimentally studied the effect of asphaltene inhibitors by injecting CO_2_ mixed with TiO_2_, SiO_2_, MgO, and graphene oxide (GO) as DAIs (direct asphaltene inhibitors) into crude oil. The results of the static test are shown in Table 3, and the use of nanoparticles as inhibitors of direct asphaltenes was reduced. Asphaltene precipitation using 100 ppm GO decreased from 5.8 wt% to 3.1 wt%, which was more effective at reducing the amount of asphaltene precipitation than other DAIs considered in this study. The authors believed that the specific surface area of GO (890 m^2^/g) is higher than that of TiO_2_ (174.5 m^2^/g), SiO_2_ (590 m^2^/g), and MgO (300 m^2^/g). Therefore, it led to more asphaltene adsorption. Induchoodan et al. [101] studied how the incorporation of graphene oxide (GO) nanoparticles affects the structural stability of asphaltene aggregates in asphalt. The author studied the GO–asphaltene aggregates. From the results of Figure 9, it can be seen that the precipitated sample contains a large agglomeration structure, and the surface adsorbs smaller particles, as shown in Figure 9a. Figure 9c shows that these particles with a particle size less than 100 nm have different contrasts compared with the agglomerates. Therefore, according to this preliminary observation, it can be predicted that the large block structure seen in Figure 9a is a graphene oxide sheet.

**Figure 10:**
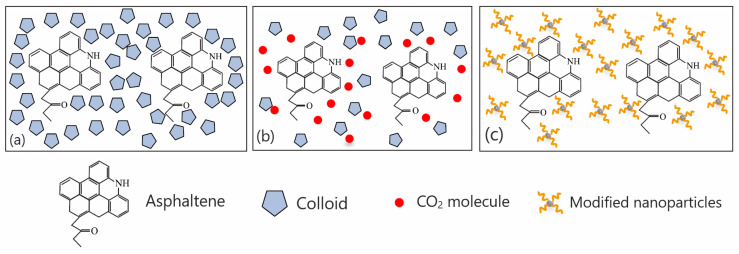
The schematic diagram of nanoparticle dispersion inhibiting asphaltene: (**a**) in a stable state, colloids form a solvation layer around asphaltenes; (**b**) CO_2_ injection destroys the solvation layer; and (**c**) nanoparticles form an alkyl space stable layer on the surface of asphaltenes. Taken with permission from [70].

In the study of organic nanoparticles, Thao et al. [102] modified the surface of iron oxide nanoparticles by introducing organic long-chain acrylic acid and 2-acrylamido-2-methylpropanesulfonic-acid-containing polar groups, which not only retained the characteristics of nanoparticles themselves, but also had strong polarity. The modified nanoparticles have the ability to form a strong interaction or steric hindrance with asphaltene. As shown in Figure 10, when the nanoparticles are prepared as a nanofluid suspension, are injected into the reservoir, and enter between the resin and the asphaltene, they will bind to the asphaltene through dipole interactions, charge transfers, and hydrogen bonding. Then, an alkyl space stable layer is established on the surface of the asphaltene molecule, thereby dispersing the asphaltene and avoiding the self-association of the asphaltene, thereby stabilizing the asphaltene so that it cannot be deposited.

Carbon quantum dots (CQDs) are usually defined as small carbon nanoparticles with a particle size of less than 10 nm and various surface passivation modifications [103]. The composition and structure of CQDs determine their different characteristics. There are heterogeneous hydroxyl and carboxyl groups on the surface of CQDs, which can promote the capture of holes by surface passivation and other methods. Therefore, CQDs have good water solubility and biocompatibility [104]. They have shown great application potential in improving oil recovery by changing the wettability, reducing the oil–water interfacial tension, reducing the crude oil viscosity, and changing the oil–water mobility ratio [105,106,107]. At the same time, compared with nanoparticles, such as SiO_2_ and Fe_3_O_4_, CQDs have the advantage of better compatibility with the asphaltene molecule structure and are potential materials for solving the problem of asphaltene deposition.

Alemi et al. [108] synthesized a new type of carbon nanoparticles to inhibit/disperse the deposition and aggregation of asphaltenes in unstable crude oil. The experimental results show that when the concentration of carbon nanoparticles was 400 ppm, the initial time of asphaltene precipitation was delayed from 26 vol% n-C_7_ to 37 vol% n-C_7_, which was attributed to the ultra-high specific surface area of carbon nanoparticles. DLS analysis showed that the average particle size of asphaltene aggregates decreased from 1730 nm of blank oil to 255 nm after carbon nanoparticle treatment. DFT simulation results show that there is a strong hydrogen bond interaction between the functional groups of carbon nanoparticles and the active sites of asphaltene, and there is a π-π interaction between the electron cloud of the asphaltene aromatic ring and carbon nanoparticles.

Ye et al. [109] synthesized oil-soluble carbon quantum dots (HCQDs) by the one-step microwave method, with an average particle size of 2.3 nm and a size distribution between 0.5 and 4 nm. The synthesized HCQDs exhibit a complete structure, uniform particle size, and excellent solubility in oil. It was found that in the presence of CQDs, the starting point of asphaltene precipitation could be delayed from 45 V% to 54 V%, the average particle size of asphaltene aggregates could be reduced from 1200 nm to about 600 nm, and the particle size distribution range of asphaltene aggregates was significantly narrowed, which effectively improved the particle size distribution of asphaltene aggregates.

### 3.3. Inorganic Nonmetal Nanoparticles

The most widely used inorganic nonmetal nanoparticles are SiO_2_ nanoparticles. Aghajanzadeh et al. [110] pointed out that silica nanoparticles have the ability to inhibit asphaltene deposition. Compared with the initial state, the relative permeability of the oil phase of the core after asphaltene deposition decreases significantly, and the residual oil saturation increases. After treatment with silica nanofluids, the effective permeability increases, the residual oil saturation decreases, and the degree of damage to the core decreases.

**Figure 11:**
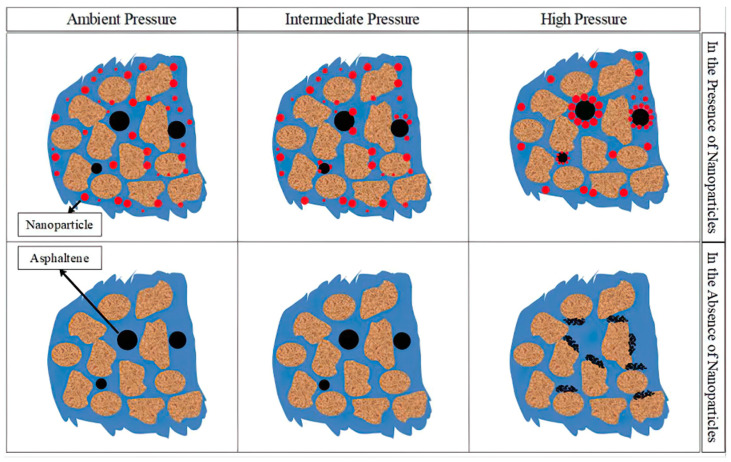
Schematic diagram of nanofluids reducing asphaltene precipitation in porous media under different pressures. Taken with permission from [111].

Kazemzadeh et al. [111] compared SiO_2_, NiO, and Fe_3_O_4_ nanoparticles, all of which can adsorb asphaltene, but SiO_2_ nanoparticles have a better effect. At the same time, as shown in Figure 11, with the increase in pressure, the number of asphaltenes precipitated and deposited in crude oil decreases because under high-pressure conditions, the wettability of nanofluids changes to a water-wet state better. Nanoparticles mainly reduce the deposition of asphaltenes by adsorbing asphaltene particles, and this adsorption increases with increasing pressure. Lu et al. [112] studied the inhibitory effect of nanoparticles (NiO, SiO_2_, and Fe_3_O_4_) on the aggregation of asphaltene particles in water-wet micropores, and found that it can effectively prevent the aggregation of asphaltene and improve the stability of asphaltene in microcapillaries. On the other hand, nanoparticles can prevent the flocculation of asphaltene particles. This is mainly due to the high surface-to-volume ratio, good adsorption capacity, and high suspension of nanoparticles. In the presence of nanoparticles, suspended asphaltene aggregates with smaller particle sizes were observed in the microcapillaries. The number of asphaltene aggregates was more than without nanoparticles, and the particle size distribution was narrower.

In addition, unlike metal oxide nanoparticles, SiO_2_ nanoparticles can not only adsorb asphaltenes but also achieve surface functionalization using grafting, thereby inhibiting the deposition of asphaltenes by dispersion. Zeinab et al. [113,114] studied synthesized silica–polyacrylamide nanocomposites and investigated their performance in inhibiting asphaltenes in unstable crude oil in water-based nanofluids. It was found that the presence of nanocomposites reduced the particle size of asphaltene aggregates. The results also show that the crude oil aggregates after nanocomposite treatment were more dispersed, and nanocomposites could improve the dispersion efficiency and dispersion of asphaltenes. The authors believed that it was the strong hydrogen bonds and London dispersion between the nanocomposites and asphaltenes that led to the successful adsorption of asphaltene aggregates, thereby inhibiting the aggregation and precipitation of asphaltenes.

### 3.4. Inhibition Effects of Different Nanoparticles

The inhibitory effect of nanoparticles on asphaltene deposition is complex. The type, concentration, preparation method, reaction temperature, and reaction time of nanoparticles have a great influence on their properties. Franco et al. [115] measured the adsorption isotherms of 12 nanoparticle asphaltenes and reservoir rocks and found that the adsorption of asphaltenes is closely related to the type of nanoparticles. The surface chemistry and morphology of nanoparticles and the chemical and physical properties of asphaltenes determine the surface adsorption of asphaltenes.

Table 4 summarizes the results of different nanoparticles applied to asphaltene adsorption. Compared with commercial nanoparticles (NPs), in situ prepared NPs have a higher viscosity before and after centrifugation, which is because they have a high degree of dispersion. At the same time, nanocomposites have a higher selectivity for asphaltene adsorption than uncoated NPs, which may be due to the increase in interaction sites and the interaction strength between asphaltene and coated NPs.

## 4. Numerical Simulations of Asphaltene Nano-Inhibitors

In addition to indoor physical experiments, numerical simulation studies on the inhibition of asphaltene deposition by nanoparticles were also gradually carried out in recent years. Bai et al. [120] selected asphaltene model molecules with heteroatoms (N, O, S) at different positions as adsorbents and studied their adsorption and desorption behavior on silica by molecular dynamics simulations. During the adsorption process, the asphaltene molecules move to the silica surface, and the asphaltene polyaromatic surface exhibits different orientations with the silica surface in the equilibrium state. Some molecules are in almost face-to-face contact with the silica surface, while others are adsorbed on the edge of the polyaromatic surface or the alkane side chain. This is mainly attributed to the balance between the asphaltene–silica interaction and π-π stacking interaction between asphaltene molecules. The numerical simulation study also pointed out that the presence of heteroatoms (N, O, S) enhances the polar interaction between asphaltenes and silica, hindering their desorption from the surface.

Mohammed et al. [121] used the classical molecular dynamics simulation method to study the structure and transport behavior of heavy multi-aromatic compounds with different structures in SiO_2_ nanopores. The simulation found that asphaltene molecules near the solid surface are first adsorbed. Then, other asphaltene molecules are adsorbed on these molecules to form dimers, trimers, or nano-aggregates at the pore entrance and pore surface. The van der Waals interaction and electrostatic interaction between the asphaltene and SiO_2_ surface are the main reasons for the adsorption of asphaltene.

Nassar et al. [122] proposed a population balance model that assumes that asphaltenes are related to aggregation and fragmentation at shear rates to describe the kinetics of asphaltene flocculation and fragmentation in the presence of metal oxide nanoparticles. As a result, under different conditions, all nanoparticles reduce the hydrodynamic radius of large aggregates in the solution to varying degrees. Varamesh et al. [123] used the cubic correlation equation of state to simulate the precipitation of asphaltene in reservoir model oil in the presence or absence of Fe_3_O_4_ and NiO nanoparticles. The simulation results show that the self-association energy of asphaltene changes exponentially with the molar density of the surface of the two nanoparticles.

Madhi et al. [124] carried out adsorption experiments on three kinds of metal oxide nanoparticles (SiO_2_, Al_2_O_3_, and MgO), and investigated the inhibitory effect of each nanoparticle on asphaltene. Four isothermal adsorption models, namely, Langmuir, Freundlich, Temkin, and Dubinin–Radushkevich, were used to analyze the adsorption data to better understand the adsorption mechanism. In these models, Dubinin–Radushkevich gave a better prediction. Mohammadi et al. [86] used the response surface methodology (RSM) to characterize TiO_2_/SiO_2_ nanofluids by combining experimental and simulation methods. The results showed that the RSM could establish a reasonable model for the delay or inhibition of the asphaltene flocculation process. When the salinity decreases, the occurrence time of asphaltene flocculation increases. At high salinity, the repulsive force decreases significantly, which increases the agglomeration rate between these particles and slightly reduces the efficiency. The observed phenomena align with the DLVO (Deryagin–Landau–Verwey–Overbeek) theory, which posits that colloidal particles primarily experience van der Waals attraction and electrostatic repulsion. The resultant force’s magnitude and direction play a crucial role in determining the stability of the colloidal system.

## 5. Field Application of Nano-Asphaltene Deposition Inhibitor

At present, the asphaltene inhibitors used in oilfields are mostly traditional organic asphaltene inhibitors, such as benzene ring surfaceactive substances, while nanoscale asphaltene inhibitors are still in the laboratory test stage. However, some oilfields have carried out nanoscale application tests.

In order to reduce the asphaltene precipitation near the well in the Cupiagua Sur oilfield in Colombia, a field test of nano-huff-and-puff was carried out in well CPSXL_4_. In this outward-facing formation, 220 barrels of nanofluid containing Al_2_O_3_ nanoparticles were injected. After eight months of operation tracking, the results showed that the asphaltene content in the produced oil of the well remained stable, and the oil production exceeded the baseline by about 300 barrels/day. By tracking and measuring the concentration of nanoparticles in the production water, it can be concluded that with the increase in operation time, the inhibition process of asphaltene deposition was effective, and the oil production remained above the baseline [125]. It can be seen that the application of nanotechnology in the Cupiagua Sur oilfield is booming, which confirms that the adsorption of asphaltene molecules by nanoparticles is effective.

Similarly, field applications were also performed in five wells in the Tenay light oil field (API 36°) in Colombia. The reservoir temperature was 220 °F and the reservoir pressure was 2300 psi. During the design operation, the nanofluid was forced into a radius of 4 feet. After a certain period (10–12 months) of operation tracking, the oil production of each well increased by about 60 barrels per day, the decline curve was significantly improved, the benefit was maintained for more than 12 months, and the total oil production increased by about 249,000 barrels. The measurement of residual nanoparticles in the production solution shows that nanoparticles with a concentration as low as 2 ppm can inhibit asphaltene precipitation. Compared with other conventional treatments, the results show a better effect [126].

Although conventional asphaltene inhibitors are mainly used to deal with asphaltene deposition during reservoir development, the inhibition effect of conventional asphaltene inhibitors tends to be greatly reduced when asphaltene deposition occurs in volatile reservoirs because the high flow rate and high pressure drop will hinder the retention of asphaltene inhibitors in the formation. Under the conditions of a specific reservoir and specific nanofluid concentration, nanoparticles can effectively inhibit asphaltene deposition, which is beneficial to further improve the oil recovery of low-permeability reservoirs. Different types of nanoparticles have different inhibitory effects on asphaltene deposition. Iron, cobalt, and nickel oxide nanoparticles can effectively adsorb asphaltene and inhibit asphaltene deposition better. Although the current research on the inhibition of asphaltene deposition by nanoparticles is still mainly focused on the indoor research stage, the successful test in the oil field shows the application potential of this technology.

## 6. Conclusions

This paper investigates the phenomenon of asphaltene deposition that may occur following crude oil system disruption, discusses the mechanism of asphaltene precipitation, and reviews three types of nano-asphaltene deposition inhibitors. The authors summarize current asphaltene inhibitors as follows for reference:

(1) The main factors that affect asphaltene precipitation are temperature, pressure, and composition. Temperature and oil composition are the main parameters that affect the stability of asphaltene. At the same time, it is believed that the colloid in the oil component mainly affects the stability of asphaltene. When the resin content is high, asphaltene is not easy to precipitate. When the resin content is low, the asphaltene becomes unstable and easy to precipitate. From the mechanism of action of the inhibitor, the nanoparticles cannot aggregate by adsorbing asphaltene molecules to inhibit precipitation; at the same time, it was shown that the grafted nanoparticles can also promote the dispersion stability of asphaltene molecules in crude oil.
(2) At present, the inhibitors with future development prospects are nano-inhibitors. These inhibitors are mainly divided into metal oxide nanoparticles, organic nanoparticles, and inorganic nonmetal nanoparticles.
  - Metal oxide nanoparticles mainly include CaO, Co_3_O_4_, and Fe_3_O_4_. These nanoparticles have attracted extensive attention due to their superparamagnetism, stability, and easy surface functionalization.
  - In the study of organic nanoparticles, the surface modification of nanoparticles is mainly used. A long organic chain containing a polar group is introduced, and it has dual properties by grafting. This not only retains the characteristics of the nanoparticles themselves, but also has a strong polarity and can form a strong interaction or steric hindrance with asphaltenes. At the same time, quantum dot nanoparticles also showed an excellent inhibitory effect on asphaltene deposition.
  - In the investigation of inorganic nonmetal nanoparticles, particularly SiO_2_ nanoparticles, they exhibit not only adsorption capability toward asphaltenes but also enable surface functionalization through grafting, thereby impeding asphaltene deposition by promoting dispersion.
(3) Nanoparticles have a larger specific surface area and adsorption capacity, have better suspension and catalytic performances, and are less likely to cause reservoir damage than chemical agents. Therefore, they have incomparable advantages over conventional organic asphaltene inhibitors in solving the problem of asphaltene deposition. In the face of asphaltene deposition in special reservoirs, such as volatile reservoirs, it also shows a more excellent inhibition effect. However, the existing nanoparticles also show the characteristics of low stability. Due to the strong interaction, the nanoparticles are easy to aggregate, resulting in a larger size and losing the expected effect. Confirming the optimal concentration of nanoparticles will reduce the key to the precipitation and deposition of asphaltenes. Excessive concentration will not only increase the treatment cost but also reduce the ability of nanoparticles to stabilize asphaltenes, resulting in an increase in asphaltene deposition and precipitation.

The authors believe that the surface functionalization of nanoparticles or the use of stable and economical surfactants to enhance their stability, as well as developing low-cost and environmentally friendly nanofluid formulations (such as nano-surfactants, nano-polymers, and nano-microorganisms) adapted to the reservoir environment, are the key to the future application of nanofluids to inhibit asphaltene deposition and improve oil recovery. In addition, the development of the complex system of nanoparticles and conventional asphaltene inhibitors and the establishment of accurate mathematical models will also be an important direction for further development.

## Figures and Tables

**Table 1:** Fe_3_O_4_, γ-Al_2_O_3_, and NiO metal oxide nanoparticle specifications. Taken with permission from [88].

Nanoparticles	Measured Size (nm)	Specific Surface Area (m^2^/g)	Chemical Nature
Fe_3_O_4_	15–20	81.98	Amphoteric
γ-Al_2_O_3_	20	>138	Acidic
NiO	10–20	50–100	Acidic

**Table 2:** Comparison of the IFT slopes of the 1st to the 2nd regions for B and N asphaltene types in the absence and presence of Fe_3_O_4_ nanoparticles at 50 °C. Taken with permission from [90].

Type of Asphaltene	Nanoparticle wt%	Slope of the 1st Region	Slope of the 2nd Region	Ratio of the IFT Slopes of the 2nd to the 1st Regions
B-Oil (5 wt%)	0	2.05	0.45	21.95%
	0.5	2.03	0.83	40.89%
N-Oil (5 wt%)	0	2.12	0.43	20.28%
	0.5	2.18	0.61	27.98%

**Table 3:** The effects of GO, TiO_2_, SiO_2_, and MgO nanoparticles on asphaltene precipitation as direct asphaltene inhibitors during a static test at reservoir conditions, 60 °C, and 3150 psia. Taken with permission from [100].

DAI	Concentration (ppm)	Asphaltene Precipitation Static Test (wt%)
Pure CO_2_		5.8
GO	50	4.0
	100	3.1
	200	2.5
	300	2.4
TiO_2_	500	5.0
	1000	4.2
	2000	3.8
	3000	3.7
SiO_2_	500	4.8
	1000	4.0
	2000	3.5
	3000	3.4
MgO	500	4.5
	1000	3.8
	2000	3.5
	3000	3.5

**Table 4:** Performance comparison of different types of nano-asphaltene deposition inhibitors.

Nanoparticles	Particles Sources	Particle Size	Adsorption Model	BET Surface Area	Asphaltene Adsorption Capacity	Other Remarks	Reference
γ-Al_2_O_3_	Commercial	<50	Langmuir	-	The adsorption capacities of the initial concentrations of 100, 500, and 1000 mg/L were 8.8, 44.6, and 68.0 mg/g.	The absorption of γ-Al_2_O_3_ by asphaltenes was spontaneous and exothermic.	[116]
Al_2_O_3_	Aladdin Reagents Co. Ltd. (Shanghai, China)	<50	Consistent with the description of type III isotherms with low adsorbate–adsorbent affinity	205.7	The adsorption capacities of the initial concentrations of 500, 1000, and 1500 mg/L were 2965, 6368, and 7857 (mg/m^2^), respectively.		[87]
		27 ± 1	Langmuir	156	Qm (mg/m^2^) values for Al_2_O_3_ at 410, 550, 700 K, and TGA were 1.4, 1.8, 2.4, and 1.7, respectively.	The shape of Al_2_O_3_ was a rod.	[117]
Alumina		Nano-alumina: 48 ± 3 nm, micro-alumina: <200 μm	Langmuir (nano), Freundlich (micro)	Nano-alumina: 39, micro-alumina: 156	Qmax (mg/m^2^): nano-alumina (1.73) > nicro-alumina (0.448).	The surface acidities of micro-alumina and nano-alumina were equivalent.	[77]
Fe_3_O_4_	Commercial	22 ± 1.5	Langmuir	43	The adsorption capacities of VBASP6, VBASP7, VBASP8, and VBASP9 were 0.047, 0.048, 0.085, and 0.197 mmol/g, respectively.	The adsorption rate increased with the decrease in the molecular weight of asphaltene.	[74]
		21 ± 2	Langmuir	43	Qm values (mg/m^2^) for Fe_3_O_4_ at 410, 550, 700 K, and TGA were 1.7, 2.2, 2.9, and 2.1, respectively.		[117]
		20–30	Langmuir	43	Qm (mg/m^2^): Fe_3_O_4_ (1.7).		[118]
Fe_2_O_3_	In situ prepared Fe_2_O_3_, commercial Fe_2_O_3_	Average diameter in nm: In situ prepared Fe_2_O_3_: 35 ± 5, commercial Fe_2_O_3_: 20–30	-	Geometrical surface area for in situ prepared Fe_2_O_3_: 25 m^2^/g	Qm values for in situ prepared Fe_2_O_3_ and commercial Fe_2_O_3_ were 2.6 and 0.9 g asphaltenes/g NPs, respectively.	In situ NPs adsorbed more asphaltene and had more selectivity for asphaltene.	[119]
		29 ± 4	Langmuir	37	Qm values (mg/m^2^) for Fe_2_O_3_ at 410, 550, 700 K, and TGA were 1.7, 2.1, 2.7, and 2.6, respectively.		[117]
NiO/SAPO-5 composite	Prepared by the sol–gel method	27.5 ± 7.5	Langmuir at high temperatures of 342 and 353 K, Freundlich at low temperatures of 298 and 328 K	304	Qm values at 298, 325, 342, and 353 K were 111.11, 109.42, 104.53, and 91.74 mg/g, respectively.	-	[92]
NiO/ZSM-5 nanocomposite	Prepared by the sol–gel method	-	Langmuir at high temperatures of 342 and 353 K, Freundlich at low temperatures of 298 and 325 K	348	Qm values at 298, 325, 342, and 353 K were 117.65, 113.64, 111.28, and 100.00 mg/g, respectively.		[93]
Fe_3_O_4_/SiO_2_; Fe_3_O_4_/TiO_2_; Fe_3_O_4_/Chitosan		Mean particle size in nm: Fe_3_O_4_/SiO_2_: 30, Fe_3_O_4_/TiO_2_: 40, Fe_3_O_4_/chitosan: 40	Brunauer–Emmett–Teller (BET)		Qm values for Fe_3_O_4_/SiO_2_, Fe_3_O_4_/TiO_2_, and Fe_3_O_4_/ chitosan were 76.50, 74.22, and 100.12 mg/g, respectively.	Nanocomposites could better adsorb asphaltene and stabilize water in oil emulsions.	[97]

## Data Availability

Not applicable.
